# Risk factors of cerebrospinal fluid leakage after neuroendoscopic transsphenoidal pituitary adenoma resection: a systematic review and meta-analysis

**DOI:** 10.3389/fendo.2023.1263308

**Published:** 2024-01-08

**Authors:** Jiahui Zhao, Shisong Wang, Xudong Zhao, Haohao Cui, Cunyi Zou

**Affiliations:** Department of Neurosurgery, The First Affiliated Hospital of China Medical University, Shenyang, Liaoning, China

**Keywords:** pituitary adenoma, cerebrospinal fluid leakage, endoscopic surgery, risk factors, meta-analysis, systematic review

## Abstract

**Introduction:**

Cerebro spinal fluid (CSF) leakage is common and might lead to severe postoperative complications after endoscopic transsphenoidal pituitary adenoma resection. However, the risk factors of postoperative CSF leakage are still controversial. This article presents a systematic review to explore the explicit risk factors of CSF leakage after endoscopic transsphenoidal pituitary adenomere section.

**Methods:**

PRISMA and AMSTAR guidelines were followed to assess the methodological quality of the systematic review. PubMed, Medline, Embase, Web of Science, Cochrane, Clinical Trails, CNKI, CBM, Wan Fang, and VIP databases were searched for all studies on postoperative CSF leak risk factors. The quality of the included studies was assessed by the Newcastle-Ottawa scale. Review Manager 5.4 software was used to calculate the pooled effect size of potential factors with statistical significance.

**Results:**

A total of 6775 patients with pituitary adenoma across 18 articles were included, containing 482 cases of postoperative CSF leakage (accounting for 7.11%). All of the articles had a quality score > 5, indicating good quality. Meta-analysis showed that an increased risk of CSF leak was found for higher levels of BMI (MD=1.91, 95% CI (0.86,2.96), bigger tumor size [OR=4.93, 95% CI (1.41,17.26)], greater tumor invasion (OR=3.01, 95% CI (1.71, 5.31), the harder texture of tumor [OR=2.65, 95% CI (1.95,3.62)], intraoperative cerebrospinal fluid leakage [OR=5.61, 95% CI (3.53,8.90)], multiple operations [OR=2.27, 95% CI (1.60,3.23)].

**Conclusion:**

BMI, multiple operations, tumor size, tumor invasion, hard texture, and intraoperative cerebrospinal fluid leakage are the risk factors of postoperative CSF leakage. Clinical doctors should pay attention to these risk factors, and conduct strict skull base reconstruction and careful postoperative management.

## Introduction

Nowadays, neuro-endoscopic transsphenoidal surgery (TSS) is the most optimal operation approach for pituitary adenoma (PA) (1–3). With the destruction of the skull base bone and cerebral dura mater, postoperative cerebrospinal fluid (CSF) leakage frequently occurs (4).Referring to past literatures, there are different reports on the incidence of CSF leakage after endoscopic transsphenoidal resection of pituitary adenomas, respectively 2.05% in 2002 by Cappabianca (5), 0.5%-15.0% in 2003 by Shile (6), 12.1% in 2011 by Messer (7). Although the probability of CSF leakage has gradually decreased with the progress of skull base repair techniques and materials, CSF leakage is still considered as the toughest problem after endoscopic transnasal surgery. CSF leak is often associated with various complications, including headache, meningitis, intracranial infection, and CSF hypotension syndrome, which may increase the long-term and cost of hospitalization and affect the prognosis of patients (8). Thus, identifying postoperative CSF leakage risk factors is particularly important for early intervention.

With increasing attention to post operative CSF leakage, more and more studies have focused on exploring the risk factors of postoperative CSF leakage. BMI, tumor invasiveness, intraoperative cerebrospinal fluid leakage, and multiple operations are universally recognized as possible risk factors for postoperative CSF leakage. Nevertheless, many studies have inconsistent or even opposite conclusions. Patel’s research suggested that tumor size was not a risk factor for postoperative CSF leakage. However, Peng’s research suggested that tumor size was the most important factor affecting postoperative cerebrospinal fluid leakage. Moreover, most of the past research was single-center retrospective studies lacking a large sample. These studies’ inclusion and exclusion criteria were different, such as surgical approach and tumor type. Slot et al. reported the overall rate of postoperative CSF leakage after TSS for PA was 3.4% in 2022, and cavernous sinus invasion and intraoperative CSF leakage were independent risk factors (8). However, this study included PA patients by endoscopic approach and a microscopic approach. Due to the rapid development of endoscopic technology, most pituitary tumors can be resected with endoscope, so it is meaningful to study the cerebrospinal fluid leakage after endoscopic surgery. In a word, there is still no comprehensive and unified conclusion on the risk factors of CSF leakage after endoscopic surgery for pituitary tumors.

Here, we conducted a meta-analysis and systematic review with an all-around literature search and comprehensive analysis to clarify the risk factors of CSF leakage after neuro-endoscopic transsphenoidal pituitary tumor resection, providing guidance for clinical treatment and prognosis improvement.

## Materials and methods

PRISMA and AMSTAR guidelines were followed in the systematic review and meta-analysis.

### Search strategy

The databases of Embase, Medline, PubMed, Web of Science, Clinical Trials, and Cochrane were searched as English literature sources. The databases of CNKI, CBM, Wan Fang, and VIP were searched as Chinese literature sources. A combination of MeSH terms and free words for postoperative CSF leak of TSS, “Neuroendoscope” OR “Transsphenoidal” AND “Pituitary Neoplasms” OR “Pituitary Adenomas” AND “Cerebrospinal fluid leak” OR “Cerebrospinal Fluid Leakage” OR “Cerebrospinal fluid rhinorrhea” AND “Risk Factors” OR “Factors”, were used to form a search string. The search was completed in May 2023.

### Inclusion criteria

(1) Study type: All the included literature were retrospective and observational studies, including cohort studies and case-control studies (2). Study object: The patients diagnosed with pituitary adenoma underwent endoscopic transsphenoidal surgery. Postoperative CSF Leak was defined as theCSF leak from the nasal cavity confirmed by biochemical and imaging examinations from operation to post operative 30 days (3). Publication date of search articles: To provide the latest literature and reduce study bias, we screened the literature from 2008 to 2023 (4). Evidence-based medicine required comprehensive inclusion of literature. Chinese studies should not only meet the requirement of a NOS score greater than 5 but also be published in Chinese core journals with national funding projects (9).

### Exclusion criteria

(1) Study type: Meta-analysis, systematic evaluation, randomized controlled study, and case report were excluded (2). Study object: Patients underwent craniotomy or microscopical transnasal surgery. Patients pathologically diagnosed as non-pituitary adenoma after surgery, such as craniopharyngioma, Rathke cyst, and so on (3). Study quality: A study with a score of less than 5 referring to the Newcastle-Ottawa scale, or a study with incomplete data and too small sample number (N<20) was excluded (9).

### Study indicators

We extracted study indicators that might affect postoperative CSF leakage from the collected literature, such as age, gender, BMI, tumor size, operation duration, operator experience, preoperative radiotherapy and chemotherapy, preoperative drug treatment, intraoperative CSF leakage, history of operation, and so on.The data of patients with these factors was collected and analyzed.

### Literature quality evaluation

The Newcastle-Ottawa scale was used to evaluate case-control and cohort studies, including object selection, comparability, and outcome (9). A study could be awarded a maximum of one star for each numbered item within the Selection and Outcome categories. A maximum of two stars could be given for Comparability. According to the Newcastle-Ottawa scale, two independent researchers evaluated the quality of the selected literature and scored them. If two researchers had different scores for the same document, let the third researcher evaluate it. The maximum score was 9 points, and the score of more than 5 points was considered good quality. The higher the score, the better the literature quality and the less the bias. We included the literature with 5 stars or more and excluded the literature with poor quality.

### Data extraction

After evaluating the quality of the literature, two independent researchers read the full text of the selected literature. We independently extracted the basic information, study characteristics, and outcome indicators of the literature with Excel. These basic information and study characteristics included the author, country, publication time, study type, sample number, statistical data, NOS points, and so on.

### Statistical analysis

The software for data analysis was Review Manager 5.4. Mean difference (MD) was used to report continuous variables (BMI). Odd rate (OR) and 95%CI were used to report binary variables (such as intraoperative CSF leakage, and multiple operations). Q test was used to verify heterogeneity. I²<50%and P>0.05 were defined as having no heterogeneity and good consistency, with selecting the fixed effect model to calculate the OR value with the Mantel-Haenszel method. If there was heterogeneity(I²>50%and P<0.05), select the random effect model to calculate the OR value with the DerSimonian and Laird method. The subgroup analysis was used to find the source of heterogeneity. The funnel plot was used to verify publication bias. If the included literature did not meet the meta-analysis criteria, qualitative analysis would be conducted.

## Result

### Literature selection and basic information

**Figure 1:**
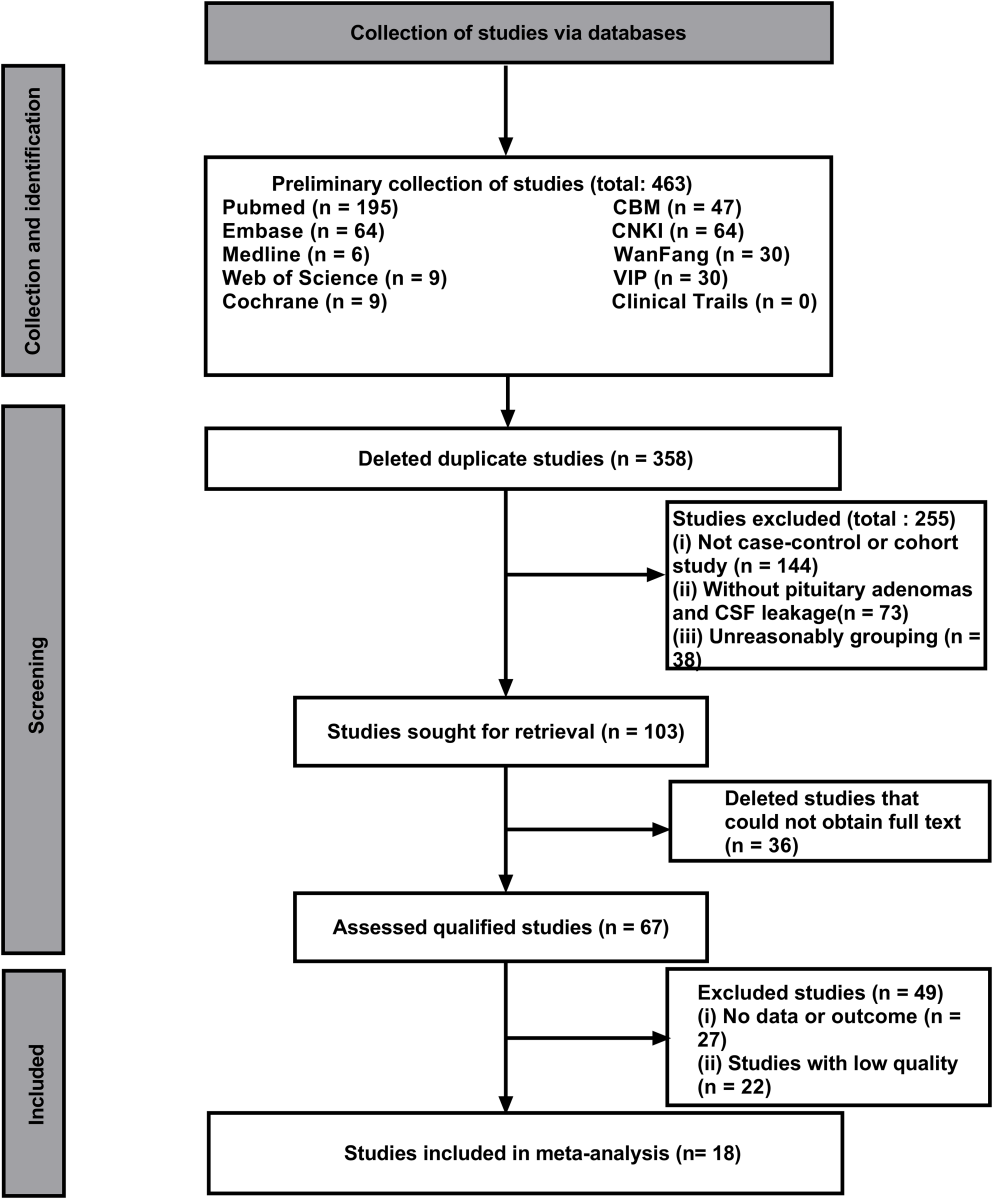
Literature research and selection chart.

In this study, 463 literatures were initially searched. After browsing and exclusion, 18 literature (14 case-control studies and 5 cohort studies) were included in the study (**Figure 1**). The basic information, such as authors, risk factors, and quality score were shown in **Table 1** (10–27).The NOS quality scores of included literatures were greater than 5 (**Table 2**).The literature that did not meet the meta-analysis criteria but could be qualitatively analyzed is listed in **Table 3**. A total of 6775 patients with pituitary adenoma and 482 cases of postoperative CSF leak were finally included. The overall incidence of postoperative CSF leakage was 7.11%.

**Table 1:** Summary of basic information of included studies.

Serial number	Author	Nation	Date	Total csses	Numberof postoperativeCSK leak cases	Risk factors	Study type	Quality points
1	Cetinalp etal. (10)	Turkey	2023	170	11/170(6.5%)	(a), (d),(o)	Cohort	6
2	Lietal. (11)	China	2022	400	14/400(3.5%)	(f), (i)	Case-control	7
3	Tang et al. (12)	China	2022	56	17/56(30.4%)	(d), (j)	Case-control	6
4	Luetal. (13)	China	2022	186	6/186(3.2%)	(d),(g),(j)	Case-control	7
5	Wang etal. (14)	China	2022	151	13/151(8.6%)	(h),(i)	Case-controll	5
6	Suetal. (15)	China	2021	178	23/178(12.9%)	(f),(i), (j)	Case-control	6
7	Pengetal. (16)	China	2021	250	9/250(3.6%)	(d),(i),(n)	Cohort	7
8	Cai et al. (17)	China	2021	158	39/158(24.7%)	(f),(m)	Cohort	6
9	Hannanetal. (18)	USA	2020	270	24/270(8.9%)	(h),(i),(k)	Case-control	8
10	Liuetal. (19)	China	2020	194	25/194(12.9%)	(d),(g),(i)	Case-control	6
11	Xue et al. (20)	China	2020	216	13/216(6.0%)	(f),(i)	Cohort	8
12	Zada et al. (21)	USA	2019	1153	30/1153(2.6%)	(f)	Cohort	8
13	Sunetal. (22)	Singapore	2018	123	10/123(8. 1%)	(c),(j)	Case-control	8
14	Pateletal. (23)	USA	2018	806	38/806(4.7%)	(c),(f),(l)	Case-control	8
15	Fraseretal. (24)	USA	2017	615	103/615(16.7%)	(c),(f)	Case-control	8
16	Karnezisetal. (25)	USA	2016	1161	68/1161(5.9%)	(a),(c),(f)	Case-control	7
17	Dlouhyetal. (26)	USA	2012	96	13/96(13.5%)	(a),(c),(i)	Case-control	8
18	Hanetal. (27)	China	2008	592	26/592(4.4%)	(g),(j),(k)	Case-control	7

(a) Age, (b) Gender, (c) BMI, (d) Tumor size, (e) Tumor grade, (f)Tumor invasiveness, (g) Hard texture of tumor, (h) Tumor pathological type, (i) Intraoperativecerebrospinalfluidleakage, (j) Multipleoperations, (k) Experience of the surgeon, (l) Combined with other intracranial diseases, (m)Albumin level, (n) Chronic respiratory disease, (o) Diabetes mellitus.

**Table 2:** The NOS quality evaluation of included studies.

Serial number	Study	Selection	Comparability	Outcome	Points
1	Cetinalp et al.2023 (10)	★★★	★	★★	6
2	Li et al.2022 (11)	★★★★	★	★★	7
3	Tang et al.2022 (12)	★★★	★	★★	6
4	Luetal.2022 (13)	★★★	★★	★★	7
5	Pengetal.2021 (14)	★★★★	★	★★	7
6	Suetal.2021 (15)	★★★	★★	★	6
7	Wangetal.2022 (16)	★★	★	★★	5
8	Cai et al.2021 (17)	★★★★	★	★	6
9	Hannanetal.2020 (18)	★★★★	★★	★★	8
10	Liuetal.2020 (19)	★★★	★★	★	6
11	Xue et al.2020 (20)	★★★★	★★	★★	8
12	Zada et al.2019 (21)	★★★	★★	★★★	8
13	Sunetal.2018 (22)	★★★★	★★	★★	8
14	Pateletal.2018 (23)	★★★	★★	★★★	8
15	Fraseretal.2017 (24)	★★★★	★★	★★	8
16	Karnezisetal.2016 (25)	★★★	★★	★★	7
17	Dlouhyetal.2012 (26)	★★★★	★	★★★	8
18	Hanetal.2008 (27)	★★★	★★	★★	7

**Table 3:** The information of studies included in the qualitative evaluation.

Risk factor	Author	Nation	Date	Total csses	Number of postoperative CSK leak cases	Study group	P value	Study conclusion
Age	Cetinalpet al. (10)	Turkey	2023	170	11/170 (6.5%)	Continuous	<0.001	Negative correlation with CSF leakage
	Karnezis et al. (25)	USA	2016	1161	68/1161 (6.5%)	Continuous	0.022	
	Dlouhy et al. (26)	USA	2012	96	13/96 (13.5%)	Continuous	0.004	
	Michael et al. (28)	USA	2015	98	11/98 (11.2%)	Binary	0.03	
Tumor size	Peng et al. (14)	China	2021	250	9/250 (3.6%)	Continuous	<0.001	Positive correlation with CSF leakage
	Lee et al. (29)	China and USA	2021	38	15/38 (39.4%)	Continuous	<0.001	
Tumor pathological type	Hannan et al. (18)	USA	2020	270	24/270 (8.9%)	Rank	0.01	ACTH adenoma is the risk factor
	Lee et al. (29)	China and USA	2021	38	15/38 (39.4%)	Rank	0.027	Nonfunctional adenoma is the risk factor
Combined with other intracranial diseases	Patel. et al. (23)	USA	2018	806	38/806 (4.7%)	Binary	0.004	Hydrocephalus is the risk factor
	Yu et al. (30)	China	2014	180	25/180 (13.8%)	Binary	0.012	Sphenoid sinusitis is the risk factor
Hypoproteinemia	Cai et al. (17)	China	2021	158	39/158 (24.7%)	Continuous	0.0477	Albumin ≤ 41g/l is the risk factor
Chronic respiratory disease	Peng et al. (14)	China	2021	250	9/250 (3.6%)	Binary	0.001	Chronic respiratory disease is the risk factor
Perioperative medication	Jaman et al. (31)	USA	2019					Leuprorelin (Gonadotropin) can cause CSF leakage
	Abe et al. (32)	Japan	2020					Bromocriptine (dopamine receptor agonist) can cause CSF leakage
	Choutkaet al. (33)	Czech Republic	2018					

### Risk factors of postoperative CSF leakage

**Figure 2:**
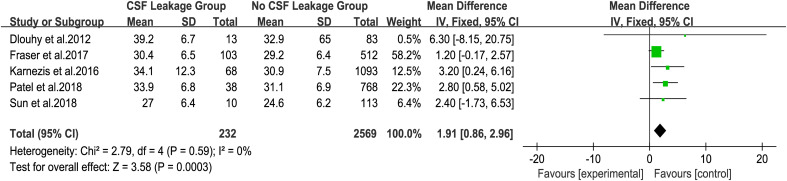
Forest plot of postoperative CSF leakage according to BMI.

To explore the influence of BMI on postoperative CSF leakage, five studies that counted BMI as a continuous variable were included to analyze (22–26). The fixed effect model analysis showed that the BMI of patients with postoperative CSF leakage was significantly higher than that of patients without postoperative CSF leakage (**Figure 2**, MD=1.91, 95%CI (0.86, 2.96), P = 0.0003). There was no heterogeneity in the five groups of studies (I² = 0%).

**Figure 3:**
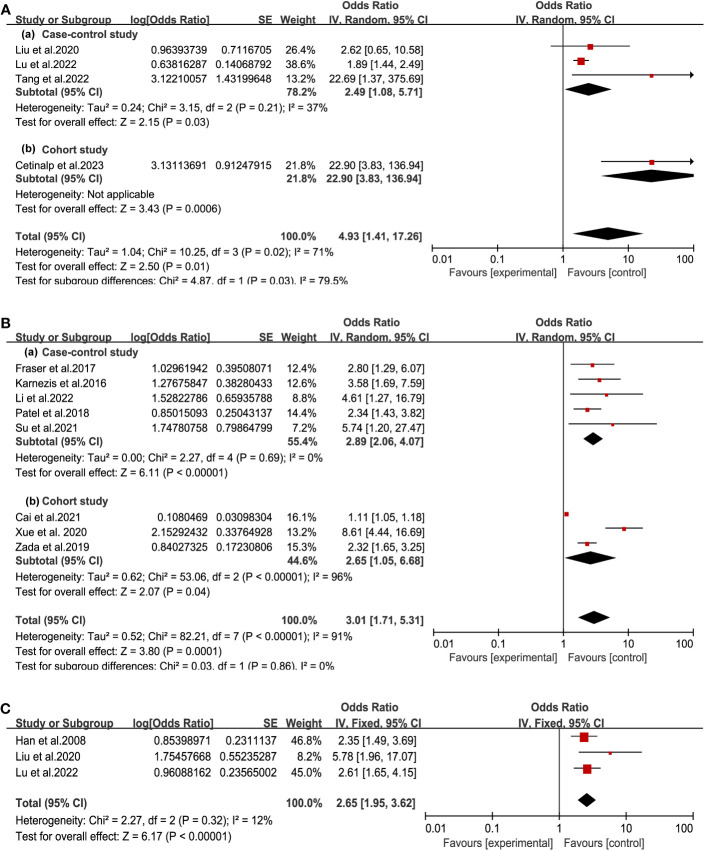
Forest plot of postoperative CSF leakage according to tumor size **(A)**, tumor invasion **(B)** and tumor texture **(C)**.

After analysis of tumor-self factors, we found tumor size, tumor invasion, and hard texture were closely related to postoperative CSF leakage (**Figure 3**). According to 4 studies among seven included articles, the tumor size was classified into a binary variable (microadenoma: d<1cm, or macroadenoma: d>1cm) (10, 12, 13, 19). Patients of macroadenoma had a higher risk of postoperative CSF leakage than microadenoma (OR = 4.93, 95% CI (1.41, 17.26), P =0. 01). Invasive tumor referred to the growth of tumor tissue beyond the intrasellar region, including suprasellar, anterior cranial fossa, posterior cranial fossa, ventricles, and other regions. Meta-analysis of 8 studies suggested invasive PA indicated a significantly increased incidence of postoperative CSF leakage (OR = 3.01, 95% CI (1.71, 5.31, P =0.0001) (11, 15, 17, 20, 21, 23–25). There was heterogeneity (I²> 50%) in the analysis of the above two factors. By grouping the cohort study and case-control study, no heterogeneity existed in the case-control study group. Furthermore, patients with a hard texture of tumors were prone to postoperative CSF leakage (OR = 2.65, 95% CI (1.95, 3.62), P<0.00001, no heterogeneity with I² < 50%) (13, 19, 27). These results indicated the smaller and less invasive tumor with earlier diagnosis could effectively reduce the occurrence of CSF leakage.

**Figure 4:**
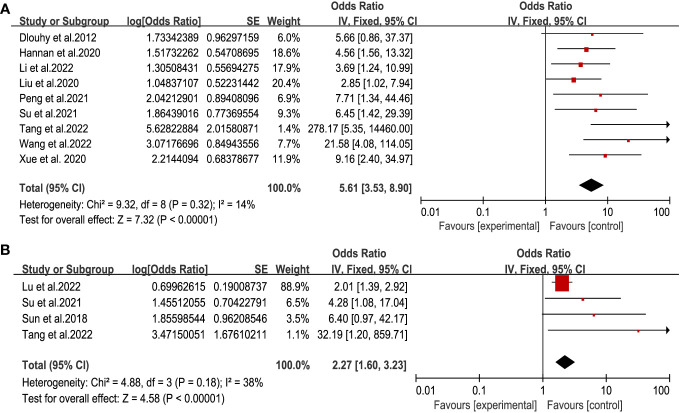
Forest plot of postoperative CSF leakage according to intraoperative CSF leakage **(A)** and multiple operations **(B)**.

The destruction of the anatomical structure of the skull base is the direct cause of CSF leakage. Considering the factors of the operation itself, we got that intraoperative CSF leakage and multiple operations were associated with postoperative CSF leakage (**Figure 4**) (11, 12, 14–16, 18–20, 26). There was significant statistical significance (respectively, OR = 5.61,95% CI (3.53, 8.90), P<0.00001, and OR = 2.27, 95% CI (1.60, 3.23), P < 0.00001) with no heterogeneity. This result highlighted the importance of skull base repair during operation.

There were not enough studies to evaluate the influence of age (10, 25, 26, 28), tumor pathological type (18, 29), and experience of the surgeon (18), combined with other intracranial diseases (23, 30), albumin level (17), chronic respiratory disease (16), and diabetes mellitus (10).We can only conduct qualitative analysis based on the literature (detailed information shown in **Table 3**).There was no significant statistical difference in the impact of gender on postoperative CSF leakage.

### Analysis of publication bias

To evaluate the bias of the six risk factors, the Egger test was performed on each risk factor. There was bias in the analysis of tumor invasion, intraoperative CSF leakage, and multiple operations (Egger test P<0.05, **Table 4**). Then, the Trim-and-fill method was performed for further evaluation. The P values before and after adjustment were all less than 0.05. These results indicated the analysis of tumor invasion, intraoperative CSF leakage, and multiple operations exist publication bias, but the conclusion was stable and reliable.

**Table 4:** The bias evaluation and the Egger test results.

Risk Factor	P Value
BMI	0.900
Tumor Size	0.125
Tumor Invasion	0.002
Tumor Texture	0.122
Intraoperative Cerebrospinal Fluid Leakage	0.004
Multiple Operations	0.007

## Discussion

Postoperative CSF leakage is one of the most common complications of neuro endoscopic TSS and has become a focus of neurosurgeons for its impact on severe adverse prognosis (21). To ensure the risk factors of CSF leakage after endoscopic transsphenoidal pituitary tumor resection, we included 6775 patients from 18 studies in recent 15 years from different languages all around the world according to the principles of evidence-based medicine. This meta-analysis through large-scale cases of retrospective studies showed that the overall incidence of CSF leakage after endoscopic TSS of pituitary adenoma was 7.11%. It is a little higher than the recent reports of 3.4% (34). BMI, multiple operations, tumor size, tumor invasion, hard texture, and intraoperative cerebrospinal fluid leakage were significant risk factors for postoperative CSF leakage in pituitary tumor patients.

### Patient’s physiological conditions

BMI is a putative risk factor for postoperative CSF leakage. When BMI increases by 5 kg/m², the probability of postoperative CSF leakage raises by 1.61 times (26). The high rate of CSF leakage after surgery in obese people might be related to the increase in intracranial pressure, for the obstruction of a cerebral venous return due to the increased pressure of the central venous, thoracic cavity, and abdominal cavity. The easier formation of arachnoid micro-thrombosis and venous sinus thrombosis leads to the increase of intracranial pressure and CSF leakage. Dlouhy et al. suggested that patients with BMI>30kg/m² should be focused on monitoring CSF leakage, and a combination of muscle, fat, and fascia materials can be used to repair the leak in patients of BMI>25kg/m² with CSF leakage (26).

The four included studies were not enough to analyze quantitatively. But young age was a risk factor for postoperative CSF leakage. This is consistent with previous literature, the younger the age, the higher the risk of postoperative CSF leakage (10, 25, 26). Also, Michael pointed out that patients over 40 years old were accompanied by a significantly lower possibility of CSF leakage (28).

Multiple transsphenoidal surgery is also a significant risk factor for cerebrospinal fluid leakage. Due to scar formation, increased tissue adhesion, vascular hyperplasia, and tissue fibrosis after the first operation, it is difficult to find and separate the residual part of the tumor during the second operation (24). More invasive anatomical operations during the resection process increase the risk of CSF leakage. Eisinge’s study also proposed a similar view, stating that fractures often lead to CSF leakage. The invasive growth tumor could erode the skull base bone (35). Multiple surgeries increase the risk of damage to the skull base structure. These all contribute to the occurrence of CSF leakage.

### Tumor factors

Tumor size, tumor invasion, and tumor texture play a crucial role in affecting postoperative CSF leakage. The macroadenoma has a wide range of wounds after resection of the pituitary adenoma, and the intracranial pressure changes greatly (36). The space left makes the arachnoid more prone to collapse, resulting in arachnoid injury and cerebrospinal fluid leakage. Meanwhile, aggressive tumors have a large adhesion area with the arachnoid membrane and have a strong erosion effect on the skull base structure (skull base bone and dura) (25). For tumors with hard texture, the operators need to pull harder and more invasive anatomical operations when removing them, leading to an increase in the probability of rupture of the dura and arachnoid membrane (27).So, preoperative MRI has been used to predict tumor texture with the T2-weighted MRI image. Usually, theT2W1 signal of the soft tumor was significantly higher than the hard one.

Whether the pathological classification of pituitary adenoma is a risk factor is uncertain. Hannan proposed that adrenocorticotropic hormone adenoma is a risk factor for cerebrospinal fluid leakage, due to centripetal obesity and higher BMI in patients with Cushing’s disease (18).While, Lee believed that nonfunctional pituitary macroadenoma was a risk factor, for the large tumor size. We need more clinical studies to confirm it (29).

### Operator factors

The operation experience of the doctors should be one of the important factors. There was a significant negative correlation between the improvement of surgical experience and the probability of postoperative CSF leakage in a retrospective study that included 270 operations performed by the same operator in 9 years (18). In the retrospective study of Messer with 473 patients, the incidence of postoperative CSF leakage in patients from 2008 to 2011was 8%. With the development of skull base repair technology and doctor experience, the probability of postoperative CSF leakage in patients decreased to 1% from 2011 to 2016 (37). The surgical experience of doctors is an important factor affecting CSF leakage in patients with pituitary adenoma after neuroendoscopic surgery, but qualitative analysis cannot be conducted based on existing literature. Moreover, if intraoperative CSF leakage occurred with the sellar diaphragm rupture, the tightness of the skull base has been compromised, resulting in postoperative CSF leakage. Patients undergoing multiple operations is also risk factor for postoperative CSF leakage. Because of scar formation and tissue adhesion in the surgery area after the first operation, it is difficult to separate the residual part of the tumor during multiple operations, increasing the risk of CSF leakage (22).

### Other factors

Patient’s own disease, such as sphenoid sinusitis, hydrocephalus, low cerebrospinal fluid protein level, and hypoproteinemia, may promote the occurrence of postoperative CSF leakage, for atrophy and ischemia of surrounding tissues and mucosa caused by inflammation, primary or secondary intracranial hypertension (16, 17, 23, 30).Obstruction of cerebrospinal fluid circulation or glymphatic clearance leads to increased intracranial pressure. Huge pituitary adenoma compresses the third ventricle before surgery leading to obstructive hydrocephalus, and it is easy to postoperative CSF leakage. Moreover, bleeding from the surgical area could cause widespread adhesion of the arachnoid membrane, resulting in CSF leakage or delayed CSF leakage (38).Besides, lower albumin levels of CSF may be a marker of the anti-inflammatory immune environment of invasive pituitary adenomas with higher infiltration of M2-like tumor-associated macrophages, which have an anti-inflammatory phenotype (39).

In addition, perioperative medication has a potential influence on postoperative CSF leakage. Emade has reported that leuprolide led to a postoperative CSF leak in a 32-year-old woman, who received hormonal fertility therapy retrieval 32 days before the presentation of her delayed CSF leak (31). Leuprolide or any drug that can potentially increase intracranial pressure should be held for 3 months after surgery or until after a skull base defect has fully healed. Considering bromocriptine resulting in spontaneous CSF rhinorrhea, especially in macroprolactinomas, patients taking bromocriptine before the operation should be vigilant for postoperative CSF leakage (32, 33).

### Skull base repair materials and techniques

Effective skull base reconstruction and repair techniques are particularly needed during operation. Kelly et al. proposed that repair should be performed according to the grading of cerebrospinal fluid leakage (40). According to the grading of CSF leakage, Grade 0 CSF leak should be repaired with a single-layer collagen sponge, Grade 1 with a double-layer collagen sponge, Grade 2 by the use of fat tissue to fill the sphenoid sinus, and Grade 3 by multi-layer method, with the collagen sponge, muscle, fat, and fascia lata or free pedicled nasal septum. Cavallo et al. innovatively proposed the 3F (Fat, Flap, and Flash) technology in 2019. They used fat to fill the dural rupture and nasal septal flap to repair the skull base structure in patients with cerebrospinal fluid leakage during surgery. Combined with early patient mobilization out of the bed following surgery, they achieved very good repair results (41).Fascia lata and free pedicled nasal septum are widely used in cerebrospinal fluid leakage repair surgery, but the pedicled nasal septum repair may cause postoperative nasal bleeding and olfactory dysfunction, and it is difficult to obtain materials in reoperation. The fascia lata avoids these shortcomings, so we recommend using it for repair (42).Muscle tissue and fibrinogen glue are now rarely used in skull base repair surgery for muscle tissue is prone to necrosis and liquefaction (43).

### Perioperative lumbar drainage

Perioperative or intraoperative lumbar drainage is also considered to be a protective factor for postoperative CSF leakage, which can reduce the probability of postoperative CSF leakage. Tan’s meta-analysis discussed the influence of intraoperative lumbar drainage on postoperative CSF leakage. The studies, including 678 cases, found that the probability of postoperative cerebrospinal fluid leakage in the lumbar drainage group was significantly lower than that in the control group (44).Alharbi proposed that lumbar drainage can be performed on patients with a potential risk of CSF leakage after surgery. If no CSF leakage occurs, remove the drainage tube within 48 hours. If there is the CSF leak, it should be drained for 3-7 days. Repair surgery should be performed if lumbar drainage exceeds two weeks (36).Meanwhile, for patients with postoperative CSFleakage exceeding 48 hours, antibiotics should be added to prevent meningitis, which is serious complication of CSF leakage to prolong hospital stays (45, 46).These suggest that effective lumbar drainage can be performed to reduce the risk of postoperative CSF leakage.

### Limitations of this study

There are some limitations to the present meta-analysis. First, all included articles were retrospective studies and lack of detailed clinical case information. So many risk factors analyzed could not do more specific grouping for statistical analysis, such as tumor size and tumor invasion. Second, due to the included studies from different countries and races, mainly USA and China, besides different experiences of neurosurgeons and researchers, these might cause clinical and methodological heterogeneity, although we have conducted corresponding heterogeneity analysis. Third, although 18 studies with 6775 patients were retrieved, the period was 15 years. With the rapid development of endoscopic technology and the improvement of repair techniques in CSF leakage surgery, the present incidence of postoperative CSF leakage should be lower than that calculated in this article. The conclusionof the meta-analysis should be updated as new original studies carry out.

## Conclusions

Through meta-analysis and qualitative analysis, the overall incidence of CSF leakage after neuro-endoscopic transsphenoidal pituitary tumor resection is 7.11%. BMI, tumor size, tumor invasion, the texture of tumors, multiple operations, and intraoperative CSF leakage are definite risk factors. Age, tumor pathology type, perioperative medication, other intracranial diseases, and hypoalbuminemia may be potential risk factors that require further study and validation. Clinicians should assess the probability of CSF leakage in pituitary tumor patients, and pay more attention to intraoperative skull base repair and postoperative management to reduce the incidence of CFS leakage and promote postoperative recovery.

## Data availability statement

The original contributions presented in the study are included in the article/supplementary material. Further inquiries can be directed to the corresponding author.

## Ethics statement

The studies involving humans were approved by The First Affiliated Hospital of China Medical University. The studies were conducted in accordance with the local legislation and institutional requirements. Written informed consent for participation was not required from the participants or the participants’ legal guardians/next of kin in accordance with the national legislation and institutional requirements.

## Author contributions

JZ: Conceptualization, Formal Analysis, Investigation, Methodology, Validation, Visualization, Writing – review & editing, Data curation, Project administration, Software, Supervision, Writing – original draft. SW: Data curation, Writing – review & editing. XZ: Data curation, Software, Writing – review & editing. HC: Data curation, Writing – review & editing. CZ: Writing – review & editing, Conceptualization, Formal Analysis, Investigation, Methodology, Resources, Validation, Visualization.
